# Quantification of Protein Secretion from Circulating Tumor Cells in Microfluidic Chambers

**DOI:** 10.1002/advs.201903237

**Published:** 2020-04-24

**Authors:** Lucas Armbrecht, Ophélie Rutschmann, Barbara Maria Szczerba, Jonas Nikoloff, Nicola Aceto, Petra S. Dittrich

**Affiliations:** ^1^ Department for Biosystems Science and Engineering Bioanalytics Group ETH Zurich Mattenstrasse 26 Basel CH‐4058 Switzerland; ^2^ Department of Biomedicine Cancer Metastasis Lab University of Basel and University Hospital Basel Mattenstrasse 28 Basel CH‐4058 Switzerland

**Keywords:** breast cancer, circulating tumor cells, CTCs, G‐CSF, microfluidics, protein secretion

## Abstract

Cancer cells can be released from a cancerous lesion and migrate into the circulatory system, from whereon they may form metastases at distant sites. Today, it is possible to infer cancer progression and treatment efficacy by determining the number of circulating tumor cells (CTCs) in the patient's blood at multiple time points; further valuable information about CTC phenotypes remains inaccessible. In this article, a microfluidic method for integrated capture, isolation, and analysis of membrane markers as well as quantification of proteins secreted by single CTCs and CTC clusters is introduced. CTCs are isolated from whole blood with extraordinary efficiencies above 95% using dedicated trapping structures that allow co‐capture of functionalized magnetic beads to assess protein secretion. The patform is tested with multiple breast cancer cell lines spiked into human blood and mouse‐model‐derived CTCs. In addition to immunostaining, the secretion level of granulocyte growth stimulating factor (G‐CSF), which is shown to be involved in neutrophil recruitment, is quantified The bead‐based assay provides a limit of detection of 1.5 ng mL^−1^ or less than 3700 molecules per cell. Employing barcoded magnetic beads, this platform can be adapted for multiplexed analysis and can enable comprehensive functional CTC profiling in the future.

## Graphical abstract

Circulating tumor cells (CTCs) isolated from liquid biopsies carry valuable functional information about the tumor phenotype. Here, a uniquely integrated microfluidic device for efficient isolation of CTCs from whole blood and subsequent determination of the protein expression as well as secretion of G‐CSF, a key factor involved in the recruitment of myeloid cells, is presented.

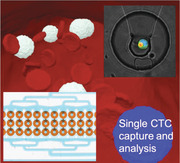

## 1 Introduction

The metastatic cascade of epithelial cancers is a complex biological process that occurs with a vast dynamic and kinetic diversity across different cancer types.^[^ ^1^ ^]^ In recent years, particular interest has been directed to the analysis of circulating tumor cells (CTCs).^[^ ^2^, ^3^, ^4^, ^5^, ^6^ ^]^ CTCs are cancerous cells that shed from a tumor site and enter the circulatory system.^[^ ^7^, ^8^ ^]^ Even though these cells are extremely rare compared to normal blood cells and have to survive a variety of stress factors while in circulation, they greatly contribute to metastasis in various cancer types.^[^ ^9^, ^10^ ^]^

In recent years, the mechanism of cancer metastasis as well as the role of the immune system have been elucidated in greater detail. Recent findings evidenced that primary tumor cells and CTCs can interact with different cells of the immune system, which actively supports the metastatic process.^[^ ^11^, ^12^, ^13^ ^]^ One particularly interesting observation is the recruitment of neutrophils not only into cancerous tissues but also at distant organs, helping to establish a pre‐metastatic niche that facilitates spreading of the disease.^[^ ^14^, ^15^ ^]^ Granulocyte colony stimulating factor (G‐CSF) plays a major role in neutrophil recruitment to distant sites, and it has recently been shown to promote cancer metastasis in certain breast cancer subtypes.^[^ ^16^, ^17^ ^]^ CTCs were found to promote the expression of G‐CSF in vivo, which in turn leads to the recruitment of neutrophils to distant sites.^[^ ^18^ ^]^ Additionally, a recent study described CTC‐neutrophil clusters as the most aggressive subset of CTCs.^[^ ^19^ ^]^ Even though these clusters are rare, these findings could affect clinical decisions as G‐CSF is currently administered as a drug accompanying chemotherapy to help counteract the effects of neutropenia.^[^ ^20^ ^]^

For personalized cancer treatment, it is crucial to understand the features of each cancer patient in detail. CTCs, circulating‐free DNA, and extracellular vesicles that are shed from cancer cells are valuable sources of information about the tumor and have hence been proposed as targets for diagnostic tests.^[^ ^21^, ^22^ ^]^ To date, the majority of analytical techniques use advanced sequencing tools to infer potential drug susceptibilities from DNA or RNA profiling.^[^ ^23^ ^]^ However, recent studies have shown poor correlation between results gained from these measurements and disease outcome or drug response.^[^ ^24^ ^]^ Therefore, direct protein analysis of CTCs may be an important asset to understand the biological processes related to drug susceptibilities in more detail.

Today, the majority of CTC isolation technologies rely either on affinity‐based capture techniques or on physical parameters such as cell size.^[^ ^25^ ^]^ Affinity‐based capture techniques target membrane markers such as epithelial adhesion molecule (EpCAM), human epidermal growth factor receptor 2 (HER‐2), or combinations thereof. Magnetic nano‐ or microparticles functionalized with antibodies targeting these receptors have been widely used for positive isolation of CTCs^[^ ^4^ ^]^ and further advanced by use of miniaturized separation or detection methods.^[^ ^26^, ^27^, ^28^, ^29^, ^30^ ^]^ Functionalization of microchannels and implementation of additional patterns or herringbone‐like topology allowed for efficient affinity‐based immobilization of CTCs.^[^ ^29^, ^30^ ^]^ Likewise, microfluidic methods employing filter structures for the capture of large CTCs from the red blood cells in whole blood have proven useful as they do not require prior knowledge on the cells of interest, are not biased to phenotypic restrictions such as expression of a surface marker, and CTCs that have undergone phenotypic changes can be captured as well.^[^ ^31^, ^32^ ^]^ Further label‐free microfluidic methods exploit hydrodynamic forces present in spiral channels,^[^ ^33^ ^]^ size‐dependent deterministic flow pathways in pillar arrays (so‐called deterministic lateral displacement),^[^ ^26^, ^34^ ^]^ microfluidic vortices generated in micro‐reservoirs aside the channel,^[^ ^35^ ^]^ or simply the inertial migration of cells in a multi‐flow microfluidic system.^[^ ^36^ ^]^ These methods have in common that the blood sample can be processed at high flow velocity in the range of mL h^−1^ and therefore, CTCs from volumes of a few mL can be enriched within a few hours (we refer to Table S1 in the Supporting Information for an overview). Besides these methods, a device for direct intravascular retrieval of CTCs has been recently presented.^[^ ^37^ ^]^

The analysis of CTCs after isolation is in most cases limited to immunostaining of membrane‐bound molecules or manual picking and combined with off‐chip DNA or RNA sequencing. Culturing of CTCs for functional testing of the CTC drug response remains challenging due to inherently low success rates.^[^ ^38,^, ^39^ ^]^ An integrated system for the measurement of matrix metalloprotease 9 secretion from CTCs was achieved through a combination of vortex CTC capture and droplet microfluidics.^[^ ^40^, ^41^ ^]^ However, washing procedures can hardly be implemented in droplet microfluidics and currently restrict the method to the analysis of enzymatic targets.

Overall, most methods for quantification of both intracellular and secreted proteins from CTCs require manual transfer of isolated CTCs to a second analytical instrument.^[^ ^42^, ^43^ ^]^ From a clinical perspective this requires not only manual work, but also might introduce measurement bias or harm the CTCs prior to analysis and thereby alter the results. In addition to the previously mentioned technological hurdles, it is unclear if isolated CTCs survive short‐term culture with conventional cell culture settings, i.e., culture medium and oxygen concentration. Even though some studies report efficiencies above 50%, the success rate of CTC cultures is generally low and only few CTC‐derived cell lines have been established to date.^[^ ^44^, ^45^ ^]^ A method that allows for the isolation of CTCs and a subsequent analysis without any further transfer or treatment would overcome the above‐mentioned bottlenecks.

Here, we present a method that allows integrated size‐selective capture and functional analysis of CTCs on a single microfluidic platform. We achieve clear distinction between CTCs and other blood components by determining the presence of HER‐2, EpCAM, and the white blood cell (WBC) marker CD45. In addition, we exploit bead‐based immunoassays that recently paved the way to quantitative analysis of proteins secreted by a few or even single cells, when used in combination with microfluidic technology.^[^ ^46^, ^47^, ^48^, ^49^, ^50^ ^]^ The unique design of our microdevice facilitates co‐capturing of barcoded beads with the isolated CTCs to quantify protein secretion from individual CTCs. We characterized the performance of the microfluidic system and performed a highly sensitive immunoassay to quantify G‐CSF secretion on cancer cells and mouse model‐derived CTCs.

## 2 Results

### 2.1 Design of the Microfluidic Device

**Figure 1:**
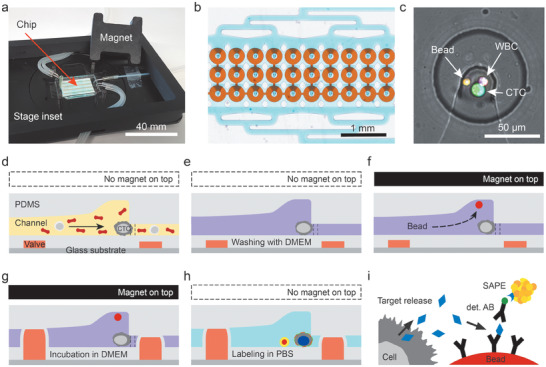
Microfluidic chip design and operation for CTC capture and analysis. a) The measurement setup consists of the microfluidic chip, the chip holder that can be mounted on the microscope stage and the lid with a permanent magnet. The photograph also shows tubes that establish fluidic and pneumatic connections to the syringe pump and pressure system, respectively. b) Micrograph of a subset of 30 analysis chambers. Each chip holds in total 1152 CTC analysis chambers arranged in four segments, each with three rows of 96 chambers. c) Zoom into the center of one analysis chambers, where size‐selective trapping of CTCs and magnetic trapping of beads are realized. Here, a CTC‐WBC cluster together with a single magnetic bead was captured and stained. The two visible light gray lines are micropillars that aid CTC capture in the cell trap. d–h) Schematics of the workflow; dashed lines indicate positions of micropillars. d) First, CTCs are captured from whole blood at the fluidic constrictions in the center of the chambers. After a successive e) washing step, magnetic beads are supplied and co‐immobilized with the CTCs. f) Therefore, the lid with the magnet is placed on top of the microchip to attract the magnetic beads. g) Next, the valves are actuated to form the analysis chamber with a volume of 80 pL. During incubation, secreted cytokine G‐CSF is bound to anti‐G‐CSF antibodies on the bead surface. Finally, the chambers are washed and labeling is conducted. h) After final washing, the microfluidic chip can be imaged. The magnet is not required during this time. i) Schematics of the sandwich immunoassay employed to detect G‐CSF.

We developed a microfluidic double‐layer polydimethylsiloxane (PDMS) device to isolate and analyze CTCs from whole blood (**Figure 1**a). A thin PDMS membrane separates the two layers, the fluid layer and the layer for valve actuation. The top fluid layer contains a channel network with 1152 trapping units for co‐capture of cells and beads. These units are arranged in four parallel segments with 3 rows and 96 columns each for reduced processing times of the 6.5 mL whole blood sample (Figure 1b). The CTCs or CTC/cell clusters are captured based on their larger size compared to other blood components through a reduction in heights of the channel after the trapping unit from 25 to 7.5 µm in combination with two micropillars forming a 2D constriction (Figure 1c). CTCs are captured while red and white blood cells can pass and are reliably flushed out of the device without clogging the channels. Occasionally, WBCs are captured, and in very few cases of <5% WBCs are co‐captured with CTCs.

After subsequent washing, magnetic beads are co‐captured in the elevated regions atop each cell trap (height of 30 µm), once a magnet is placed on top of the microfluidic chip. This ensures close proximity of the beads and the cells to capture the secreted factor efficiently (Figure 1d–j).

The bottom control layer contains pneumatic donut‐shaped valves isolating the cells and the beads into small ≈80 pL chambers when a pressure of 2 bar is applied. We ensured complete closure of fluidic channels with a multilayer photolithography process that yields a smooth transition between channel‐structures of different heights (see Figures S1 and S2 and Tables S2 and S3, Supporting Information).

### 2.2 Cell Capture Efficiencies

**Figure 2:**
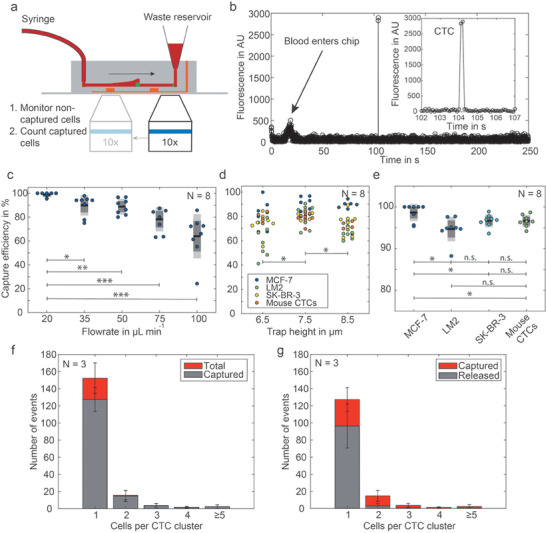
Determination of the CTC capture efficiency. The schematic in (a) shows the workflow to detect first the noncaptured cells at the outlet by recording fluorescence microscopy images, and subsequently taking images of the analysis chambers to count the captured cells. For these measurements, the cells are either stained with calcein AM or express cytosolic GFP. b) Fluorescence trace obtained by analysis of the recorded images at the outlet. c) Capture efficiency at different flow rates. d) Comparison of the capture efficiency for different channel trap heights at constant flow rate of 50 µL min^−1^; n.s. *p* ≥ 0.05; *p < 0.05, **p < 0.001, ***p < 0.0001. e) Capture efficiency for various cell types (trap height: 7.5 µm, flow rate: 20 µL min^−1^). f) Individual capture efficiency for single MCF‐7 cells and cell clusters of different sizes. g) Release of captured CTCs by applying an inverse flow of 1000 µL min^−1^ PBS with 1% BSA for 1 min (*N* refers in all graphs to the number of independent experiments on different microdevices).

For the analysis of CTCs, it is essential that the cells can be captured at high efficiency from whole blood. Therefore, we first evaluated the capture performance of the device. Once the PDMS chip was mounted on the magnetic holder (Figure S3, Supporting Information), it was primed with 20 µL phosphate buffer saline (PBS) supplemented with 1% bovine serum albumin (BSA). Thereafter, we flushed a 100 µL healthy donor blood sample that was spiked with 50 cells of the tested cell line into the device at varying flow rates. As the cancer cells were stained with calcein AM, we could detect noncaptured cells on their flow path to the chip outlet (**Figure 2**a). Therefore, a channel section of 1.2 mm by 0.7 mm, corresponding to a volume of 21 nL, was imaged every 10 ms. Even at the maximum tested flow rate of 100 µL min^−1^, noncaptured cells were detected on at least two subsequent images (Figure 2b). After the entire sample volume was processed, the chip was washed with 100 µL Dulbecco's modified Eagle medium (DMEM) medium supplemented with 10% fetal bovine serum (FBS) to remove residual blood components. Then, the capture section of the device was imaged to count the number of trapped cells. The capture efficiency was defined as the ratio between captured cells and the sum of captured and noncaptured cells.

We measured the influence of the flow rate on the capture efficiency of MCF‐7 cells in devices with a gap size of 7.5 µm. We found decreased capture efficiencies from 98.6% to 68.0% with increasing flow rates from 20 to 100 µL min^−1^ (Figure 2c, and Figures S4 and S5, Supporting Information). The optimum capture efficiency was found at a flow rate of 20 µL min^−1^. At this flow rate, a 6.5 mL patient sample is processed in 325 min. Next, we varied the gap height and found that a height of 7.5 µm performed better than gaps of 6.5 or 8.5 µm with *p*‐values of 0.015 and 0.019, respectively (Figure 2d). As MCF‐7 cells are particularly large in contrast to many other cancer‐derived cell lines, capture efficiencies were additionally tested for the breast cancer cell lines SK‐BR‐3 and MDA‐MB‐231 LM2 (LM2) as well as LM2 CTCs derived from a mouse xenograft model (Videos S2 and S3, Supporting Information). All model cells yielded average capture efficiencies above 95%; the lowest capture efficiency of a single sample was 88% measured with LM2 cultured cells (Figure 2e). Figure 2f depicts the dependency between the size of trapped cell clusters and the capture efficiency for the model cell line MCF‐7 at a flow rate of 50 µL min^−1^.

Finally, we analyzed the success rate of releasing cells after capture. Retrieval of captured cells from the device was only possible with inverse flow rates of 1000 µL min^−1^. This is 20‐fold higher than the blood processing and exerts high shear forces and elevated pressure on the cells. We found that CTC clusters have a 4.5‐times lower release efficiency than single CTCs, i.e., 17% for CTC clusters compared to 76% for single CTCs, and the overall release efficiency was only 67% (Figure 2g). This finding underlines that on‐chip analysis of captured cells is preferential to the option of cell release and off‐chip testing as it avoids loss of the rare CTCs.

### 2.3 Cell Viability after Capture

To confirm that CTCs can survive the cell culture conditions on the chip after capture, we tested the cell viability of different cells, namely, MCF‐7, SK‐BR‐3, LM2, and CTCs from LM2 xenografts on the chip. After capture, the cell viability was observed for up to 8 h at 37 °C at 85% humidity (Figure S6, Supporting Information), while the valves are closed or opened. Since all but the SK‐BR‐3 cells express cytosolic green fluorescent protein (GFP), the viability was monitored by measuring the fluorescence intensity and evaluating the morphology of the cells. The SK‐BR‐3 cells were stained with calcein AM before use.^[^ ^51^ ^]^ For the LM2 cells, a viability of 93.48 ± 1.36% was observed after 8 h of incubation when the valves were open. With closed valves, this decreased to 89.84 ± 0.80%. For LM2 xenograft CTCs, the viability was monitored for 4 h and resulted in viabilities of 96.75 ± 1.01% and 95.33 ± 2.15% with open and closed valves, respectively (Figure S7, Supporting Information). An additional 13 h long experiment with LM2 xenograft CTCs performed on one chip resulted in a 92.47% viability. For both cell types, we observed occasional cell attachment to the BSA‐coated glass substrate and cell division could be observed as well (Figure S8, Supporting Information). Taken together, these results prove that the capture process does not harm the cells and that the BSA‐coated PDMS substrate is suitable for cell proliferation. Besides, the 80 pL chamber volume is sufficient to maintain the isolated cells for the measurement time.

### 2.4 Optimization of the Immunoassay for G‐CSF

For the analysis of the secreted factors, we co‐immobilize magnetic beads functionalized with antibodies at the trapping site. After the cells were trapped on the device, fresh medium was flushed through the chip and magnetic beads were introduced. We used 6.5 µm Luminex barcoded magnetic beads pre‐coated with surface‐bound primary antibodies against human G‐CSF.

**Figure 3:**
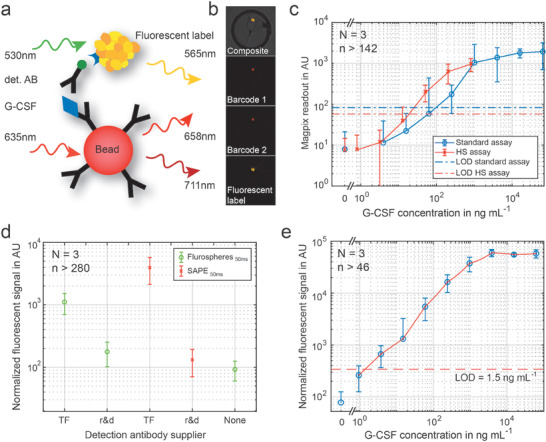
Optimization of the G‐CSF assay on fluorescently barcoded magnetic beads. a) Schematics of the sandwich immunoassay, which is performed on magnetic beads functionalized with the capture antibody. The biotinylated detection antibody (det. AB) is tagged with a fluorescent label. We finally chose streptavidin‐conjugated phycoerythrin (SAPE). b) Fluorescent signal on one bead trapped in a single microchamber. We used barcoded beads to unambiguously identify and localize the bead. c) G‐CSF sandwich immunoassay performed in microwell plates. Two different assays from TF were tested, the standard and the high sensitivity (HS) assay. The limit of detection is defined as the mean of the control measurement (*c*_G‐CSF_ = 0 ng mL^−1^) plus three times the standard deviation of the control. d) Comparison of the detection antibody from the two different suppliers, both tagged either with SAPE or fluorospheres from TF. e) Final calibration curve for G‐CSF used for the quantification of the G‐CSF production of CTCs with reduced variation and a low detection limit of 1.5 ng mL^−1^, corresponding to ≈3700 molecules per chamber. (*N* refers to the number of different microfluidic chips used for obtaining data from *n* different chambers per chip).

Two G‐CSF assay kits are commercially available from Thermo Fisher (TF) and r&d biotechne (r&d). Both kits consist of five main components. These components are the solution of magnetic beads, the lyophilized protein standard, the biotinylated detection antibody, and a solution of the streptavidin‐phycoerythrin (SAPE) label (**Figure 3**a,b). The kits were found fully functional on the 96 well plate level with limits of detection (LOD) in the lower ng mL^−1^ range, but provided high LOD when the signals of individual beads were analyzed instead of averaging several hundred beads from one well. The LOD was determined as the mean of the background signal (test with 0 ng mL^−1^ G‐CSF) plus three times its standard deviation and is 100 ng mL^−1^ in the standard assays and 15 ng mL^−1^ in the high sensitivity assay (Figure 3c). Hence, none of the commercial kits could be directly employed for on‐chip experiments. We therefore optimized each step of the assay individually. First, we chose the antibody‐coated beads from the TF kit, as it provides a five times higher bead concentration, which translates into a higher fraction of 75.8 ± 1.3% occupied chambers per chip in bead capture experiments (Figure S9, Supporting Information). Next, different combinations of detection antibody and fluorescent labels revealed the highest signals for the detection antibody from r&d compared to the one from TF (Figure 3d). Finally, the SAPE label provided by TF is an order of magnitude brighter than the label by r&d, and is even brighter than streptavidin‐labeled Fluospheres of 40 nm diameter (Figure 3d). A combination of the best performing components from the two suppliers resulted in a final limit of detection of 3678 molecules, achieved in the 80 pL chambers (Figure 3e). Noteworthy, we imaged the entire microfluidic chip with a standard 20 × (NA = 0.75) objective (see Table S4 in the Supporting Information for optical configuration), allowing us to assess the quantitative information of G‐CSF in all chambers within 50 min.

### 2.5 Quantification of Single‐Cell G‐CSF Secretion and EpCAM and HER‐2 Expression

**Figure 4:**
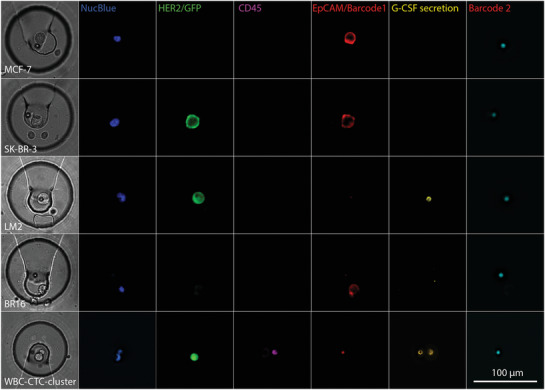
Brightfield (first column) and fluorescence images of the trapping site, occupied by individual cells of the investigated cell lines, and for comparison, a trapped WBC (bottom row). The pseudo‐colored fluorescent images reveal the presence of a nucleated cell (NucBlue) and the presence or absence of the membrane proteins HER‐2, CD45, EpCAM as well as G‐CSF secretion captured on the magnetic bead. The bead is identified by its fluorescence ratio at 658 nm (barcode 1)/712 nm (barcode 2).

After characterization and optimization of the microfluidic method, we employed our system to investigate the expression profiles of HER‐2, EpCAM, and G‐CSF of several breast cancer cell lines. After cell capture and washing, 5 µL of the magnetic bead stock solution was infused at a flow rate of 10 µL min^−1^. Once the beads reached the trap section of the chip, the cover with the permanent magnet was placed on top of the PDMS microchip to attract the beads and trap them in close proximity to the isolated cells. We washed the chip once more with 50 µL DMEM cell culture medium at 10 µL min^−1^ and actuated the valves to isolate co‐captured cells and beads for an incubation time of 4 h. During incubation, the surrounding channel was continuously flushed with medium at 1 µL min^−1^. Following incubation, all chambers were opened and washed at 10 µL min^−1^ for 5 min, before labeling was conducted in two steps using an antibody cocktail and the SAPE solution. First, a mixture of NucBlue, biotinylated G‐CSF detection antibody, anti‐EpCAM Alexa 647, anti‐CD45 PerCP, and anti‐HER‐2 Alexa 488 was supplied for 30 min at a constant flow of 0.2 µL min^−1^. After washing with 50 µL DMEM medium, the SAPE label was introduced for another 30 min at 0.2 µL min^−1^ to bind to the detection antibodies. This was followed by another washing step. Last, the entire trapping area was imaged with a 20× air objective with NA = 0.75 and a Hamamatsu Orca Flash camera (**Figure** **4**). Based on these fluorescence images, we could simultaneously identify cells and beads in each microchamber. The fluorescent signals enabled us to count all nucleated cells, differentiate CTCs from CD45 positive WBCs, retrieve the expression levels of HER‐2 and EpCAM, and quantify the G‐CSF secretion with the sandwich immunoassay that co‐localizes with the fluorescent signal of the magnetic bead.

**Figure 5:**
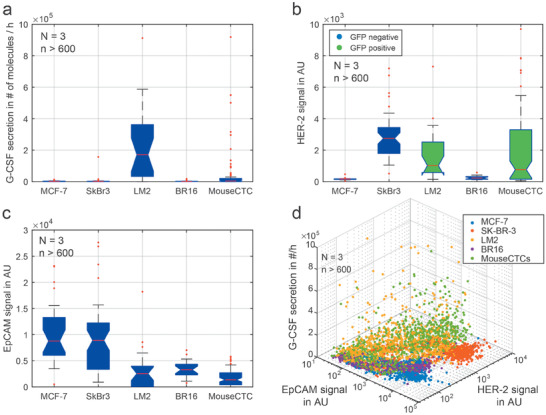
Protein expression of single cells and mouse model CTCs. a) Quantitative analysis of the G‐CSF secretion levels after 4 h of incubation. b) Comparison of HER‐2 expression for the five tested cell lines. The LM2 cells and mouse model CTCs did express GFP, which obscures the HER‐2 expression. c) Comparison of EpCAM expression in all tested cell lines. d) 3D scatterplot correlating EpCAM and HER‐2 signals with the G‐CSF secretion. This enables the discrimination of the individual cell lines. *N*: Number of microdevices, *n*: total number of analyzed cells.

Among the five investigated cell lines, we found no detectable secretion levels of G‐CSF in MCF‐7, SK‐BR‐3 and the CTC‐derived BR16 cells. In contrast, LM2 cells had a diverse phenotype with high G‐CSF expression. On average, the LM2 cells secreted 2.6 × 10^5^ G‐CSF molecules per hour, whereas LM2 xenograft CTCs had an average expression of only 8.4 × 10^4^ G‐CSF molecules per hour (see **Figure 5**a and Figure S10, Supporting Information). The expression of surface proteins HER‐2 and EpCAM is also largely different in the investigated cell lines. The HER‐2 expression was highest in the SK‐BR‐3 cells, as expected, but low for MCF‐7 and BR16 cells (Figure 5b and Figure S11, Supporting Information). As the LM2 cell line and the LM2 xenograft CTCs express low GFP levels, which overlaid with the HER‐2 signal, the HER‐2 expression level of these cells cannot be compared with the GFP‐free cell lines. Finally, EpCAM expression is highest in MCF‐7 cells, followed by SK‐BR‐3 and BR16 cells and low for the other cell lines (Figure 5c). By correlating all markers, the various cell lines can be distinguished, as indicated in the 2D plot in Figure 5d.

## 3 Conclusion

We introduced a potent microfluidic method for capturing CTCs from whole blood at a high efficiency of up to 95% for flow rates of 20 µL min^−1^ in combination with quantification of G‐CSF secretion. The highly efficient capture as well as viability of CTCs was successfully demonstrated for various cell lines including a patient‐derived BR16 CTC cell line and CTCs from LM2 xenografts. Therefore, our platform exceeds the efficiency of many existing microfluidic methods (Table S1, Supporting Information) as well as the commercial CellSearch instrument,^[^ ^52^, ^53^ ^]^ while enabling the processing of a standard full blood sample without pretreatment within 5–6 h. Further parallelization will reduce the processing time in future and automation of protocol steps such as the attachment of the permanent magnet for bead capture and improvements of the imaging software will further simplify handling of the microdevice.

In contrast to alternative methods, our platform enables the simultaneous analysis of multiple membrane proteins and secreted factors by immunohistochemistry and bead‐based sandwich immunoassays. Since we used magnetic beads that were co‐immobilized with the CTCs in miniature chambers, we were able to measure secreted molecules with unprecedented sensitivities. Further multiplexed analysis of secreted factors or intracellular proteins after cell lysis is possible due to the use of fluorescently barcoded beads.^[^ ^46^ ^]^ The isolation in 80 pL volumes as well as an optimized combination of capture and detection antibody provided the required sensitivity of the assay for the cytokine G‐CSF. The protein profiles for EpCAM, HER‐2, and G‐CSF enable differentiation between the MCF‐7, LM2, SK‐BR‐3, and BR16 cell lines. The obtained results for HER‐2 and EpCAM match previous findings obtained by RNA and protein level studies in bulk and at the single‐cell level (www.proteinatlas.org).^[^ ^42^ ^]^

G‐CSF is involved in neutrophil attraction to primary tumors and CTC‐neutrophil clusters were additionally found to be the most aggressive subset of tumor cells in circulation. Our results show that G‐CSF is secreted in isolated CTCs and the secretion levels for G‐CSF can be directly quantified. We also found CTC‐WBC clusters that consisted of WBCs with G‐CSF receptors attached to CTCs. As the G‐CSF receptors bind target molecules as well, they interfere with the bead assay. Therefore, we could not quantify the G‐CSF secretion in such cases.

Our results highlight the possibility to perform direct proteomic profiling of CTCs to gain a better understanding of the molecular pathways and signals involved in the metastatic process. By expanding this method to the analysis of other secreted proteins, we will be able to gain new insights into the underlying biological processes of cancer metastasis, which is necessary to identify new diagnostic tools and drug targets for cancer treatment in future.

## 4 Experimental Section

A list of all chemicals and reagents used in this study can be found in Table S1 in the Supporting Information.

##### PDMS Chip Fabrication

The two‐layer PDMS chips were fabricated by replica molding in PDMS from the silicon master molds (Figure S13, Supporting Information). The AutoCAD drawing is provided in the Supporting Information. The detailed protocols for silicon master and PDMS chip fabrication are appended as Tables S3 and S4 in the Supporting Information.

##### Cell Culture

MCF‐7 cells (ATCC HTB‐22) and SK‐BR‐3 cells (ATCC HTB‐30) were cultured in DMEM medium supplemented with 10% FBS at 37 °C with 5% CO_2_ and 95% humidity. MDA‐MB‐231 LM2 (LM2) human breast cancer cells (obtained from J. Massagué, Memorial Sloan Kettering Cancer Center, NY, USA) were grown in DMEM F12 medium supplemented with 10% FBS in a humidified incubator at 37 °C with 20% O_2_ and 5% CO_2_. BR16 CTC‐derived cell line was cultured as reported before.^[^ ^54^ ^]^ For a simplified detection in the mouse model, an LM2 variant cell line was created by transduction with lentiviruses carrying GFP‐luciferase (GFP) at a multiplicity of infection <5. All cell cultures were split twice a week at a 1:5 ratio for MCF‐7, a 1:2 ratio for SK‐BR‐3, and a 1:10 ratio for LM2 cells.

##### Animal Model

The mouse model was maintained in the animal facility of the University Basel, Switzerland. All mouse experiments were performed according to institutional and cantonal guidelines (mouse protocol 2781, cantonal veterinary office of Basel‐City). Immunocompromised NSG (NOD‐scid‐Il2rgnull) mice were injected with 1 × 10^6^ LM2‐GFP cells into the mammary fat pad 5 weeks prior to the experiments. For injection, breast cancer cells were inoculated in 100 µL of 50% Cultrex PathClear Reduced Growth Factor Basement Membrane Extract in PBS. They subsequently developed breast cancer and spontaneously generated CTCs and metastasis. All mice were randomized before the experiments and blindly selected before injection.

##### Experimental Setup

Before any experiment was conducted, a PDMS chip was primed by inserting pipette tips containing 20 µL deionized H_2_O in the inlet and outlet and the pressure ports, and the chip was centrifuged at 800 × *g* for 10 min. This ensured filling of the chip without residual air entrapment. Next, 1% BSA in PBS was flushed through the channels for 5 min at 10 µL min^−1^ to block nonspecific protein adsorption. The chip was then fixed onto a custom stage mounted on a fully automated fluorescence microscope equipped with an incubation chamber (see Figure S3, details in Tables S5 and S6, Supporting Information). For all experiments, the system was heated to 37 °C and a humidity control chamber was set to 100% humidity and 5% CO_2_. Due to air exchange with the surrounding atmosphere, the final humidity around the chip reached 85%. Fluid flow was applied with a neMESYS syringe pump equipped with 1 or 6 mL plastic syringes connected to the chip with polytetrafluoroethylene tubing. For actuation of the pressure valves, the four pressure ports were connected to compressed air at 2 bar and could be actuated individually using manual valves.

##### Capture Efficiency Tests

The capture efficiency of the system was assessed using human blood samples from healthy donors spiked with different cancer cell lines (MCF7, SK‐BR‐3, and LM2) and CTCs from a mouse model (LM2 xenografts). For visualization, ≈10^5^ cells from the cell culture were incubated in 1 mL of 1 × 10^−6^ m calcein AM in cell growth medium for 30 min at 37 °C prior to the experiment. GFP positive CTCs from the mouse model were spiked in whole human blood samples without additional calcein AM staining. All cells were spiked into human blood to achieve a final concentration of 500 cells mL^−1^. Blood specimens were obtained from the University Hospital Basel under the study protocols EKNZ BASEC 2016–00067 and EK 321/10, approved by the Swiss authorities (EKNZ, Ethics Committee northwest/central Switzerland) and in compliance with the Declaration of Helsinki. The capture efficiency was then tested using a sample volume of 100 µL under varying flow rates and for different gap heights. Numerical simulations to assess the pressure drop and shear rates at these flow rates were conducted to make sure that physiological levels were not exceeded (see Figure S14, Supporting Information). The outlet of the chip was continuously monitored at 100 Hz with a high sensitivity Andor iXon Ultra EmCCD camera to detect missed cells during processing of the sample. The cells were enumerated after capture by screening the locations of all traps of the chip. The capture efficiency was then defined as the fraction of captured cells over the sum of captured and noncaptured cells. For three chips, a 1 mL sample with a spiked cell concentration of 200 LM2 cells per mL was tested. The processed blood was collected at the chip outlet and the number of missed cells as well as the cluster size was determined. The results were then compared to the captured cells and cell clusters. To test the release of captured cells, CTC and cluster count were additionally determined after flushing the channel with 1 mL PBS with 1% BSA at an inverse flow rate of 1000 µL min^−1^.

##### Viability Testing

The on‐chip viability of the isolated cells was assessed both with open and closed chambers. To be sure that the time frame of the assays was covered, on‐chip viability of the cells was measured by monitoring the calcein fluorescence every 30 min for at least 4 h. The three different cell lines MCF‐7, SK‐BR‐3, and LM2 cells, as well as GFP‐transfected CTCs from the mouse model were tested.

##### Bead‐Based G‐CSF Assay Optimization

The G‐CSF bead‐based immunoassays were purchased from TF Scientific, and r&d biotechne. The binding capacity of the 6.5 µm beads was not provided by the manufacturers, but could be estimated to at least 10^7^ target molecules per bead. At first, both the assays were compared using the manufacturers’ handling protocol and thereafter the sensitivity was optimized by combining compounds from both suppliers to increase the sensitivity and allow for single‐cell studies. The assay optimization was done on transparent flat‐bottom 96 well plates using a MagPix reader and the fluorescent microscope for read‐out.

##### Single‐Cell Analysis On‐Chip

For tests with cell culture cell lines, 10 µL of a cell solution with 5 × 10^5^ cells mL^−1^ was introduced to the PDMS chip and washed with 50 µL of DMEM medium supplemented with 10% FBS. Next, 5 µL of the Luminex bead solution was inserted at a flow rate of 10 µL min^−1^ and under constant observation. As soon as the beads were seen entering the microchambers, the permanent magnet was placed on top of the chip. Next, the chip was flushed with 50 µL of DMEM medium and the pneumatic valves were closed at 2 bar for 4 h of incubation. During the incubation, secreted G‐CSF could bind to the antibody on the bead surface, while the surrounding channels were constantly flushed with DMEM F12 medium at a flow rate of 0.25 µL min^−1^ to prevent evaporation. After incubation, the flow was set to 10 µL min^−1^ and the valves were opened to wash the microchambers. In the following, 20 µL of an antibody cocktail (NucBlue, biotinylated G‐CSF detection antibody, anti‐EpCAM Alexa 647, anti‐CD45 PerCP, and anti‐HER‐2 Alexa 488 at a volumetric ratio of 82:15:1:1:1) was introduced and constantly flushed through the chip at 0.2 µL min^−1^ for 30 min as labeling times above 30 min did not result in further increase of the fluorescent signal. Binding of secreted molecules onto the bead surface was estimated with Comsol Multiphysics (Figure S15 and Video S1, Supporting Information). After another washing step with 50 µL medium at 10 µL min^−1^, the SAPE solution was introduced and incubated for 30 min at a constant flow of 0.2 µL min^−1^. After a final wash step with 50 µL of medium at 10 µL min^−1^, the valves were actuated, the permanent magnet was removed, and the whole chip was imaged.

##### Image Acquisition and Analysis

Images were acquired on a Nikon Ti microscope with a 20× objective (NA = 0.75, MRD30205) and seven different channels (see Tables S5 and S6, Supporting Information). Images of the whole chip were acquired using 2 × 2 binning in all channels to reduce the size of the image stack and improve the sensitivity. In total, a large image consisting of four individual tiles with 15% overlap was acquired in four rows with 30 positions each. The spacing between rows was set to 4.5 mm to match the chip design and the individual columns were set only 1 mm apart to overlap by 15%. As a result, images were acquired at 480 positions at the end of each experiment. Using nine fluorescent channels, this took ≈50 min on this system. For patient samples, the chip was manually screened for CTCs, and images were acquired only at the positions of successful CTC capture to reduce imaging time. Analysis of the images was then performed using NIS Elements, Fiji (ImageJ), and the final data were plotted with Matlab.

## Conflict of Interest

The authors declare no conflict of interest.

## Supporting information

Supporting Information

Supplemental Movie 1

Supplemental Movie 2

Supplemental Movie 3
