# Supplementary material for: Quantification of Protein Secretion from Circulating Tumor Cells in Microfluidic Chambers
Source: Adv Sci (Weinh). 2020 Apr 24;7(11):1903237. doi: 10.1002/advs.201903237 (PMC7284199; doi:10.1002/advs.201903237)
Supplement: Supplementary file 1 — Supporting Information [file ADVS-7-1903237-s001.pdf]

Copyright WILEY-VCH Verlag GmbH & Co. KGaA, 69469 Weinheim, Germany, 2018.

Supporting Information

Quantification of protein secretion from circulating tumor cells in microfluidic chambers

*Lucas Armbrecht, Ophélie Rutschmann, Barbara M. Szczerba, Jonas Nikoloff, Nicola Aceto, and Petra S. Dittrich**

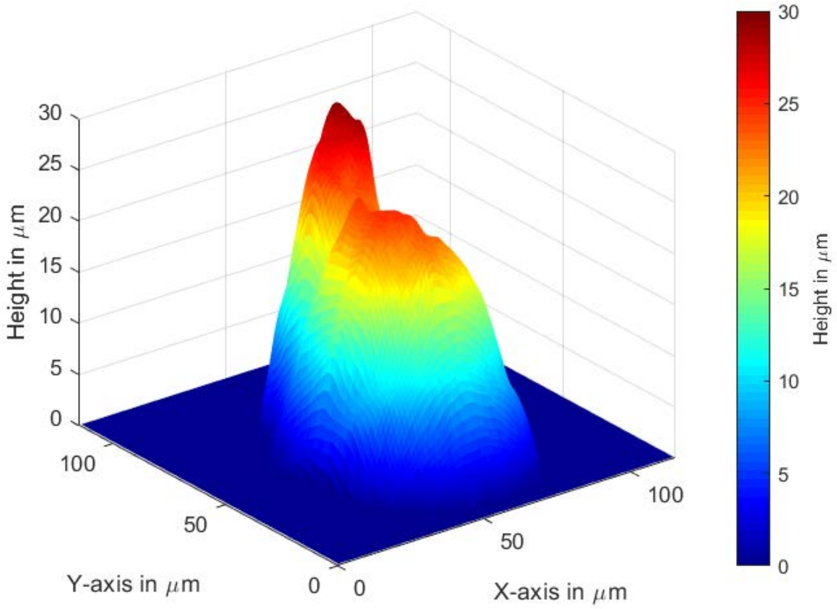

Figure S1. Size of the microchambers as determined using a profilometer and fluorescent images. The combined information of both techniques enables a precise calculation of the inner volume of the microchamber when the valves are actuated. We calculated a total chamber volume of 82 pL.

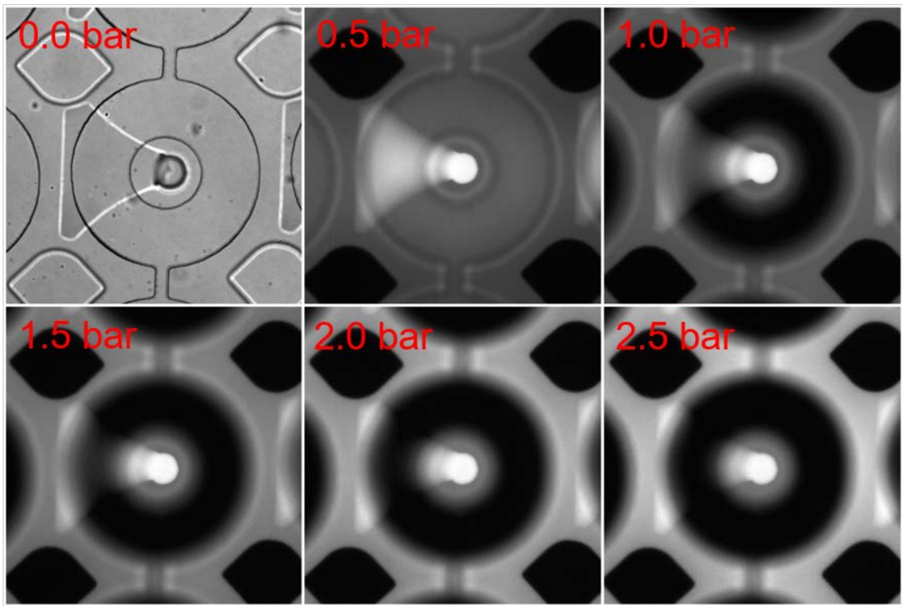

Figure S2. Actuation of pneumatic valves with different applied pressures. For complete sealing of the microchambers, a pressure above 2.0 bar has to be applied.

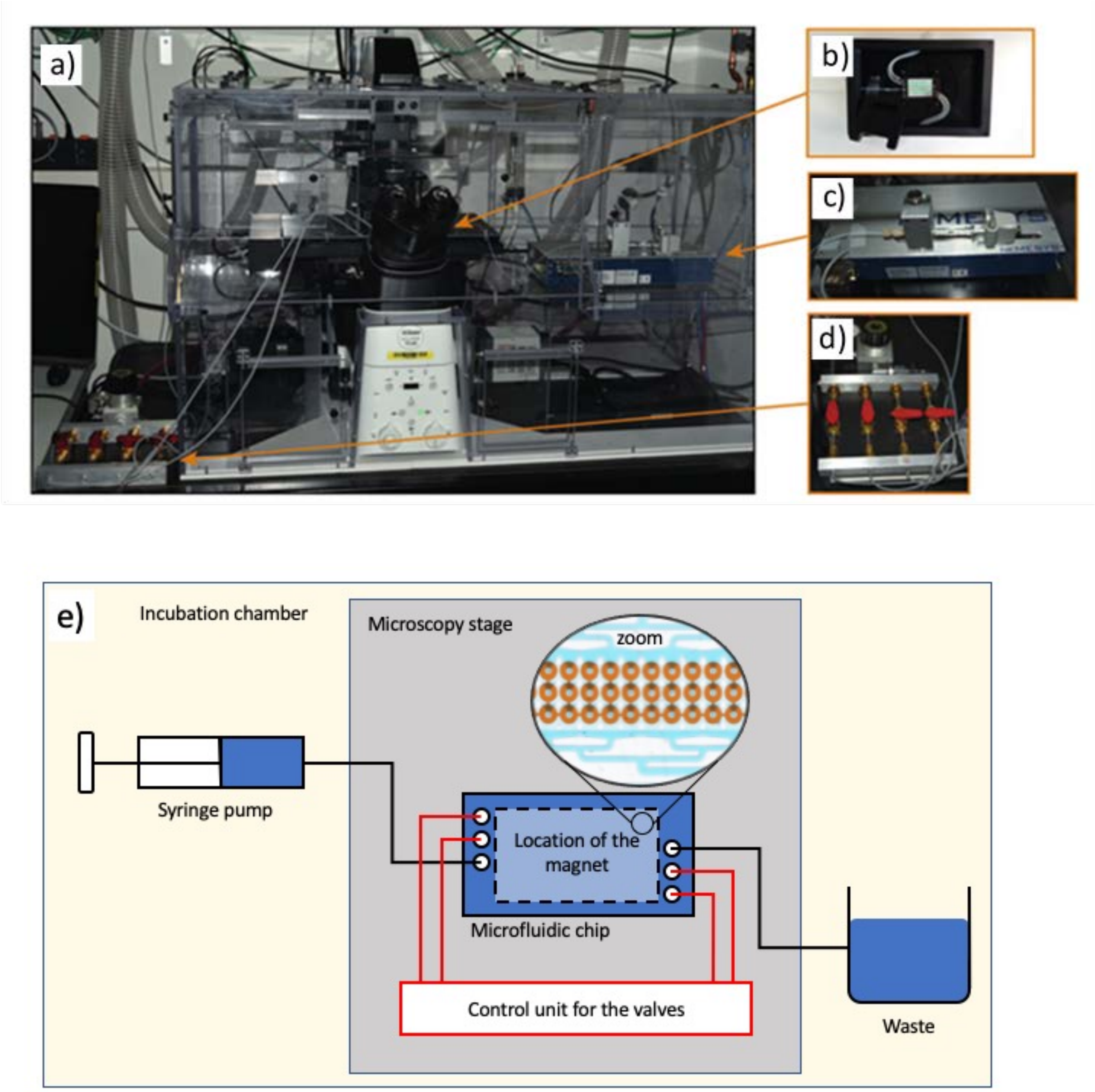

Figure S3. a) The imaging work-stations consist of fully automated inverted microscope enclosed in an environmental control box. b) The microfluidic chip is mounted onto a stage inset, which is covered by a lid to set the relative humidity around the chip close to 100% at 5% CO_2 and 37°C. c) Fluid flow is controlled with a syringe pump, the pneumatic valves are actuated through a manual valve manifold (d). e) Schematic drawing of the system. The chip has one inlet/outlet and four control lines for the valves.

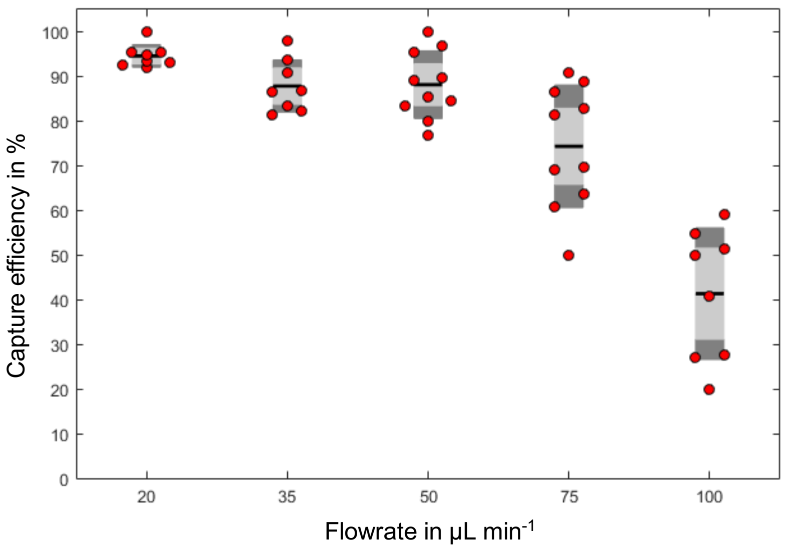

Figure S4. MCF-7 cell capture efficiency with 6.5 µm gap size for varying flow rates. Each flowrate was tested on eight microdevices.

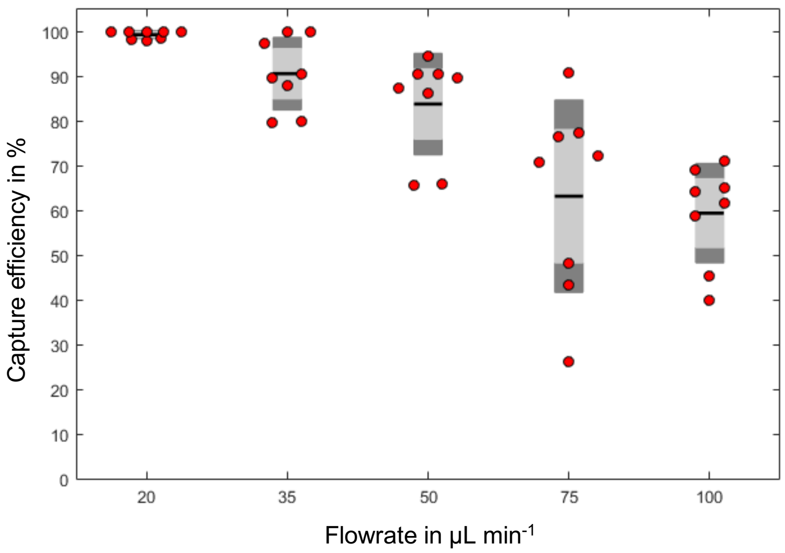

Figure S5. MCF-7 cell capture efficiency with 8.5 µm gap size for varying flow rates. Each flowrate was tested on eight microdevices.

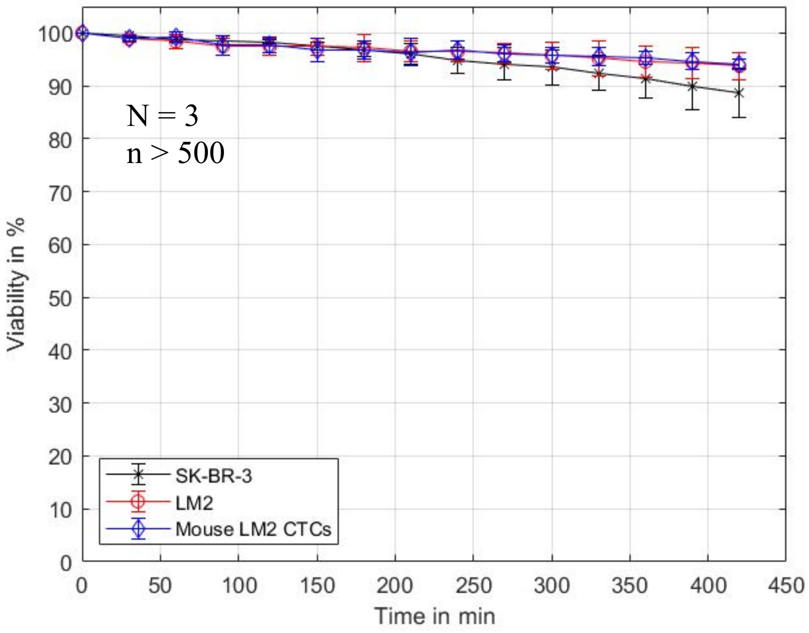

Figure S6. Viability test of isolated cells from different cell lines on the microfluidic device.

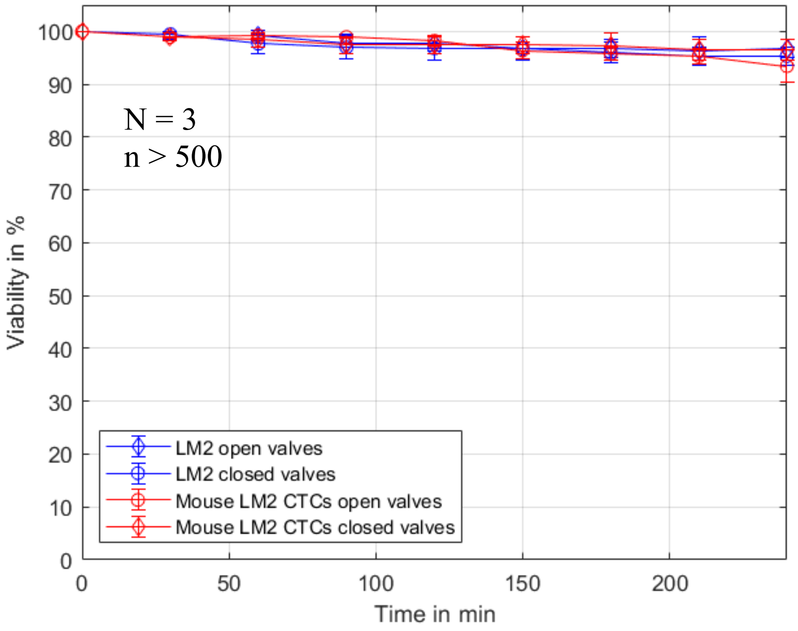

Figure S7. Viability tests on LM2 cells and mouse model CTCs to assess the influence of the limited volume in the microchambers on the cell viability. No influence was observed indicating a proper supply of nutrients for the 4 h incubation period.

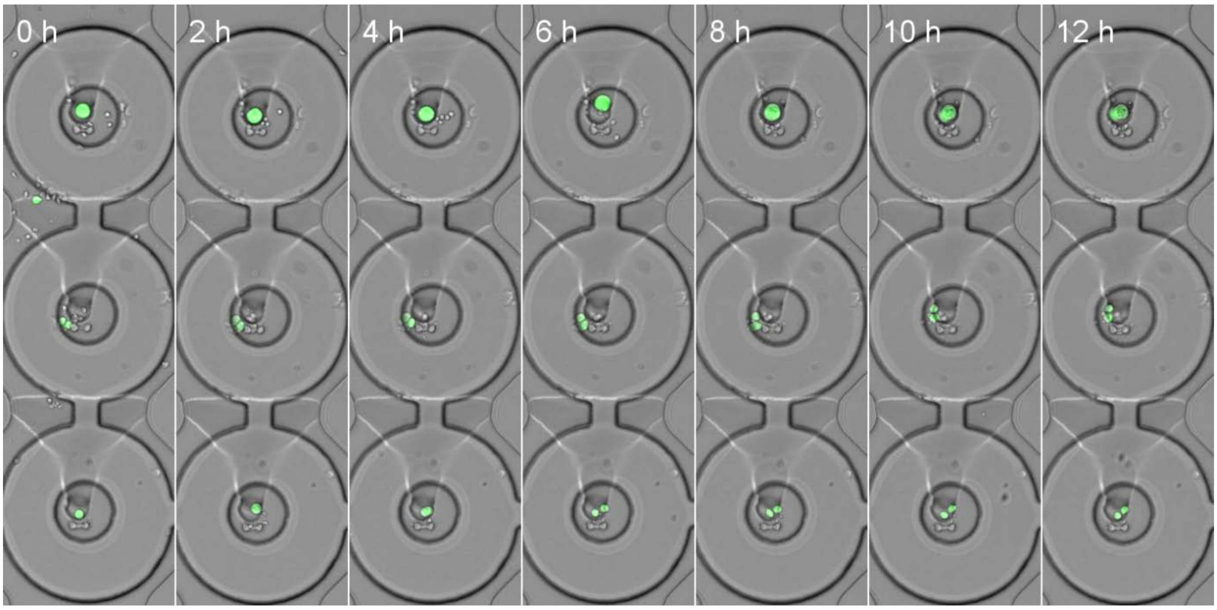

Figure S8: Time series of Mouse model CTCs (three chambers, top to bottom) incubated on chip with closed chambers. As seen in the top and bottom chamber with initially one cell, cell division was observed suggesting proper culturing conditions for the cells.

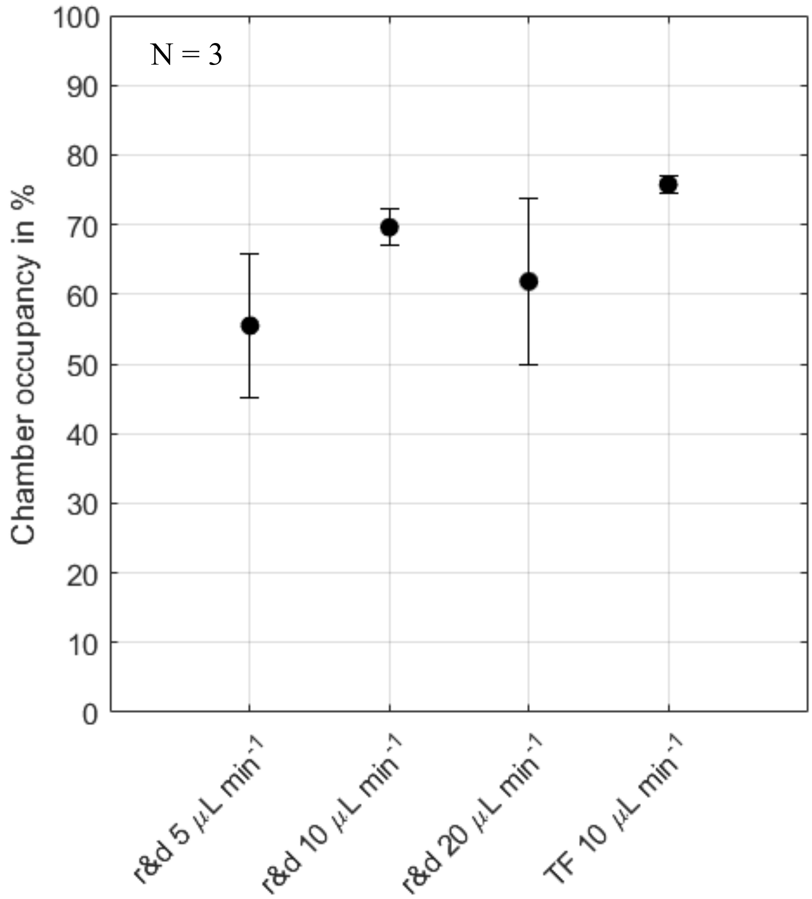

Figure S9. Capture of magnetic beads from different suppliers on the microchip.

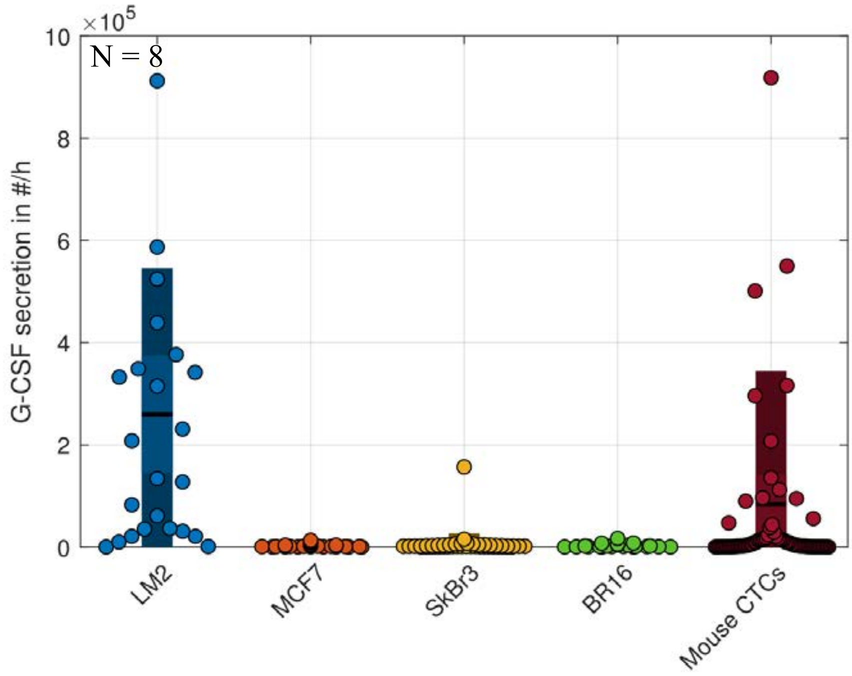

Figure S10. G-CSF secretion of all tested cell lines plotted with the individual data points for single-cell studies in the microfluidic devices. Compared to the LM2 cell line, mouse model CTCs with LM2 cell origin show a higher number of non-secreting cells.

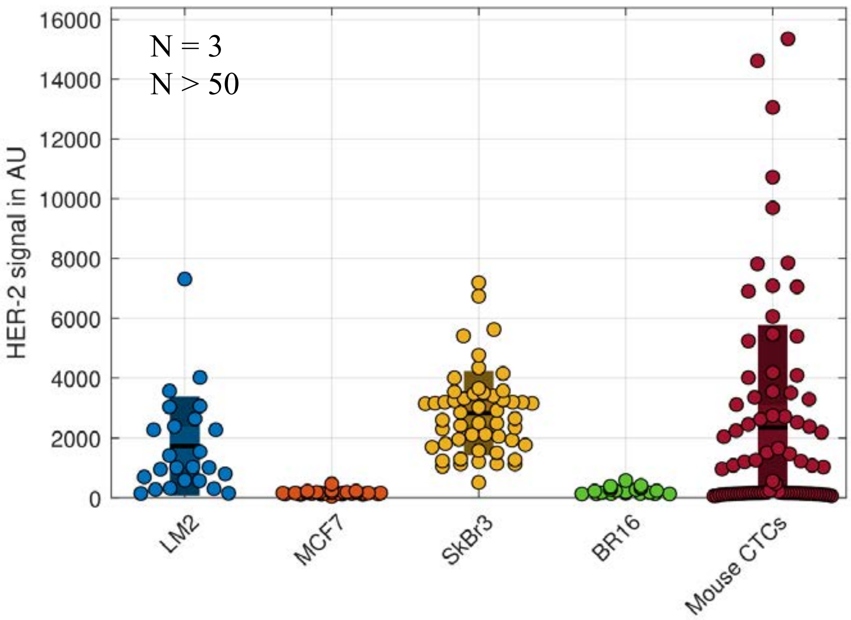

Figure S11. HER-2 secretion of the tested cell lines plotted with the individual data points for single-cell experiments in the microfluidic devices. Compared to the LM2 cell line, mouse model CTCs show a higher heterogeneity in HER-2 expression.

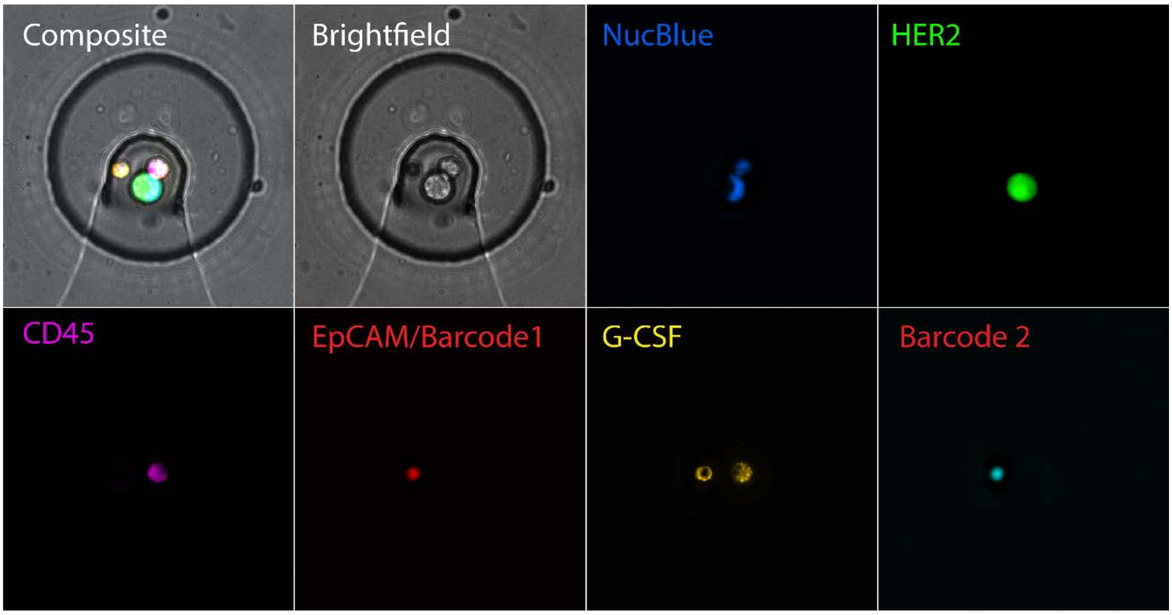

Figure S12. Pseudo-colored fluorescent images of a CTC-WBC cluster isolated from a mouse blood sample. The WBC is identified based on the CD45 expression. The signal for G-CSF detection antibody indicates that the WBC has receptors to bind G-CSF on its surface.

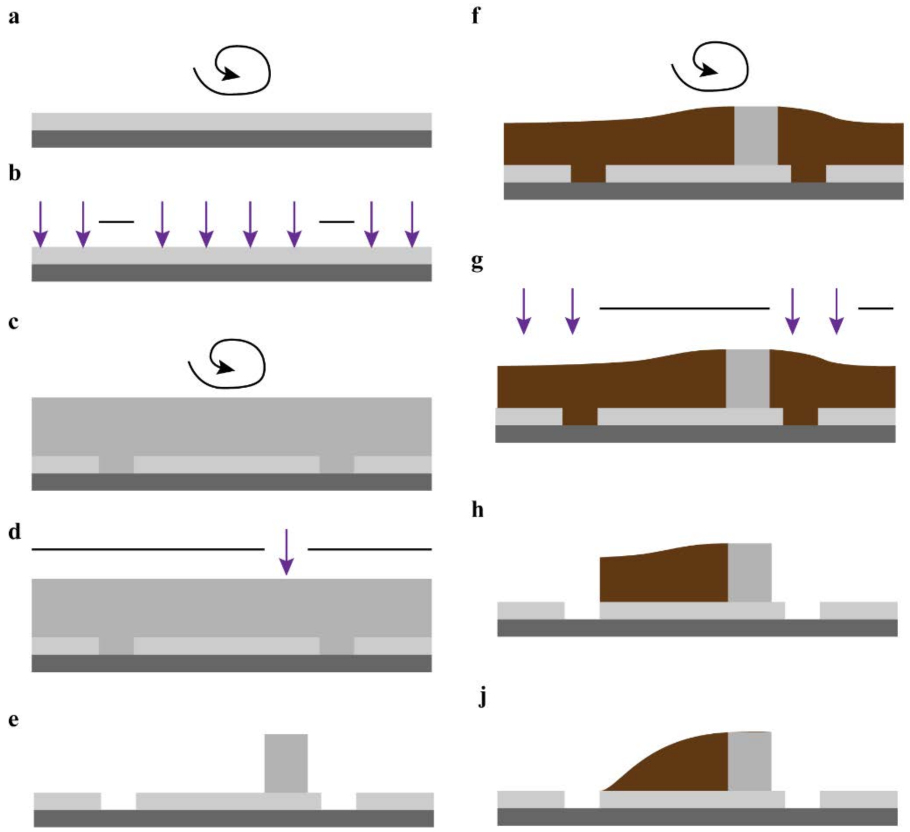

Figure S13. Fabrication routine of the silicon master mold for the fluid layer. 7.5 µm SU-8 3005 are spin-coated on the silicon wafer (a) and subsequently patterned through a foil mask (b). Thereafter, the second SU-8 3025 layer is spin-coated onto the wafer and exposed through a second foil mask (c and d). After a post-exposure bake, the SU-8 structures were developed and fixed with a hard bake (e). AZ 40XT positive resist was then spin-coated on top of the SU-8 structures with a speed that results in a lower resist height than the second SU-8 layer (f). After exposure through a third foil mask (g), the resist was developed (h) and a thermal reflow process realized the desired channel shape with smooth transitions between structures of different heights (j).

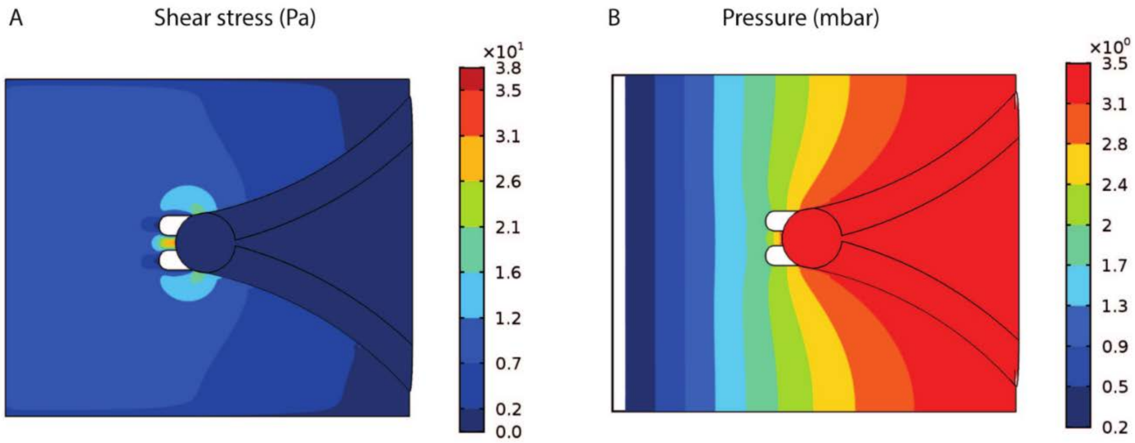

Figure S14. Simulation of shear stress (left) and pressure gradients (right) established in the microfluidic chamber, when a fluid flow of 20 µL min^{-1} is applied. The pressure drop across the whole chip is below 15 mbar, which does not exceed the pressures in circulation. (Systolic pressure is typically below 180 mbar.)

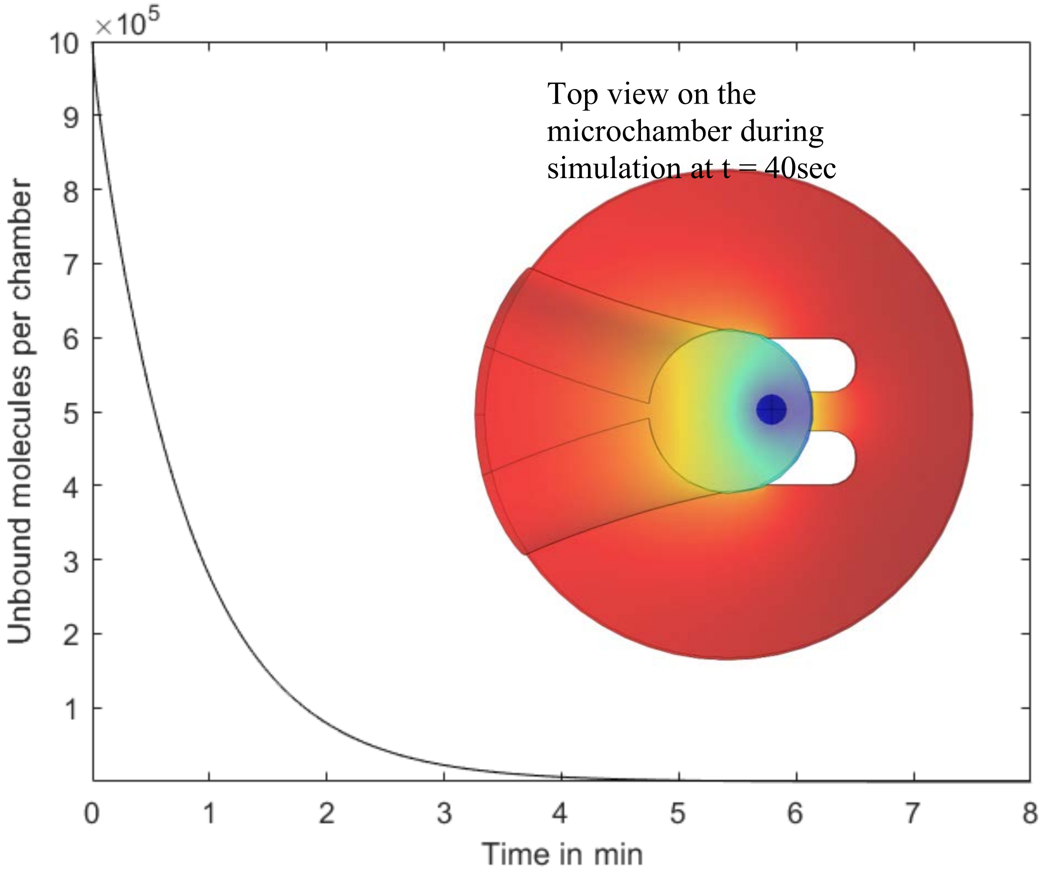

Figure S15. Binding of target molecules on a bead trapped in the microchamber over time. At time zero, homogeneous distribution of target molecules is expected. The total number of molecules in the chamber at the start was set to 10^6. The results indicate that more than 99% of the molecules are bound within 5 min. However, the simulation assumes that bound target cannot be released and that the number of capture sites on the bead far exceeds the number of target molecules. The inset depicts the simulated concentration gradient in the microchamber at time point t = 40sec. The corresponding video of the 3D diffusion simulation can be found in supplementary Video SV1.

Supplementary tables.

Table S1. Microfluidic methods for CTC capture and analysis.

**Technology**	**Capture efficiency**	**recovery**	**Processing speed for 6.5mL blood**	**Hands-on time**	**On-chip protein analysis / LOD**	**Reference**
Columns formed by magnetic beads, (surface marker CD19)	94%	-	>24h	unknown	Immunostaining	1
Immobilization on surfaces using (surface marker EpCAM), enhanced by herring-bone channel design	90%	-	>24h	unknown	Immunostaining	2
"CTC-i-chip": combination of size separation (DLD) and magnetolabelling (EpCAM)	95%	Almost 100%	2h	1.5 h	-	3
Vortex platform (size-based separation based on inertial microfluid-dics) + droplet emulsi-fication for analysis	80%	Almost 100%	10min + several hours for the functional test	unknown	Immunostainig and analysis of one secreted enzyme by a fluorogenic assay /$\sim 10^4$ enzymes	4,5
"Parsortix^{TM} system": Size selection based on "weir-type" channels	98%	50%	2h	10 min	Immunostaining	6
	42-70%	54-60%				7
Size-based separation by Dean flow fractio-nation in spiral channel	85%	Almost 100%	2h	10 min	-	8,9
**This manuscript**	Size 95%	80%	5h	1 h	Immunostaining and quantification of secretion / <3000 molecules	-

[1] Saliba, A.-E., et al. (2010). Proc. Natl. Acad. Sci. U.S.A., *107*(33), 14524–14529.
[2] Stott, S. L., et al (2010). Proc. Natl. Acad. Sci. U.S.A., *107*(43), 18392–18397.
[3] Ozkumur, E., et al. (2013). Science Transl. Med., *5*(179), 179ra47.
[4] Sollier, E., et al. (2014). Lab Chip, *14*(1), 63–77. https://doi.org/10.1039/c3lc50689d
[5] Dhar, M., et al. (2018). Proc. Natl. Acad. Sci. U.S.A., *115*(40), 9986–9991.
[6] Chudziak, J., et al. (2016). The Analyst, *141*(2), 669–678.
[7] Hvichia, G.E., et al. (2016). Int. J. Cancer, *138*(12), 2894-2904.
[8] Hou, H. W., et al. (2013). Scientific Rep., *3*, 1259.
[9] Warkiani, et al. (2014). Lab Chip, *14*(1), 128–137.

Table S2: List of chemicals and devices used for this study.

**Chemical name**	**Distributor**	**Article number**
Bead-based immunoassays		
BSA	Sigma Aldrich	A7906-50G
PBS	Thermo Fisher	14190094
Calcein AM cell-permeant dye	Thermo Fisher	C1430
G-CSF bead kit	Thermo Fisher	EPX01A-12001-901
G-CSF bead kit	r&d biotechne	LUHM214
Luminex basic kit	Thermo Fisher	EPX010-10420-901
Luminex basic kit	r&d biotechne	LUHM000
NucBlue	Thermo Fisher	R37605
Anti-HER2 Alexa 647	Biolegend	324412
Anti-HER2 Alexa 488	Biolegend	324410
Anti-EpCAM Alexa 647	Biolegend	324212
Anti-CD45 perCP	Biolegend	304025
Streptavidin Fluospheres	Thermo Fisher	F8780
Cell culture		
DMEM	Thermo Fisher	11330032
DMEM F12	Thermo Fisher	11330032
FBS	Thermo Fisher	10270106
Trypsin-EDTA (0.05%), phenol red	Thermo Fisher	25300-054
Cultrex PathClear Reduced Growth Factor Basement Membrane Extract	r&d Biosystems	3533-010-02
Chip fabrication		
4” Si wafer, <100>, p-dpot	Silicon Materials	-
SU-8 (3005/3025)	MicroChem	-
AZ 40XT (Developer)	MicroChem	-
Mr Dev 600	MicroChem	-
AZ 726MIF (Developer)	MicroChem	-
Acetone (4x2,5L ULSI)	MicroChem	-
Isopropanol (4x2,5L ULSI)	MicroChem	-
Dowsil 184 PDMS kit	Farnell	101697
trichloro(1H,1H,2H,2H-perfluorooctyl)silane	Sigma-Aldrich	448931-10G
Chlorotrimethylsilane	Sigma-Aldrich	33014
Teflon-AF	Sigma-Aldrich	469610-1G
Chemical name	Distributor	Article number
Fluorinated FC-40 oil	Sigma-Aldrich	F9755-100ML
Glass slide (50x24mm^2, #3)	Menzel B.V. & Co. KG	-
Petri dish (120x120mm^2)	Deltalab	200204
Consumables		
Biopsy puncher 1.0mm	Miltex GmbH	REF 33-31AA
Biopsy puncher 1.5mm	Miltex GmbH	REF 33-31A
1/15” PTFE tubing	PKM SA	AWG-TFT 20-N
1mL plastic syringe	BD	REF 309 628
6mL plastic syringe	Henke-Sass, Wolf GmbH	4050.X00V0
PEEK Adapter, 1/4-28 FB, F to 10-32 C, M	Ercatec AG	P-652
PEEK Adapter, F Luer to 10-32 C, F	Ercatech AG	P-659
96-well plate (glass bottom, black)	Sarstedt AG	94.6000.024
Devices		
Plasma cleaner	Harrick Plasma	PDC-002
Oven (80degC)	Memmert	100-800
Hot plates	Heidolf	MR3002
Balance	Mettler Toledo	AE240
Centrifuge 3-18K	Sigma-Aldrich	-
Mask Aligner MA6	SÜSS MicroTec AG	-
Spin coater	Laurell tech. corp.	WS-400BZ-6NPP/LITE
neMESYS syringe base unit	Cetoni GmbH	NEM-B100-01A
neMESYS syringe pump unit	Cetoni GmbH	NEM-B101-02C
neMESYS syringe pump unit	Bio-Rad	171015001

Software		
Matlab 2018a	MathWorks	-
NIS Elements 5.01	Nikon	-
neMESYS User Interface	Cetoni GmbH	-
Fiji (imageJ)	-	-
Bio-Plex Manager MP Software	Bio-Rad	-
Comsol 5.2a	Comsol Inc.	-

Table S3: Fabrication protocol for the multilayer Master structures used for replica molding of the fluid channel in the presented microfluidic device.

Step	Name	Details
1	Plasma cleaning	Plasma clean the blank SI-wafer for 5 min at 400 W to remove any residual organic substances from the silicon surface.
2	Dehydration bake	Bake the wafer for 5 min at 200°C to evaporate the hydration layer on the surface of the wafer and increase the bond between resist and wafer material.
3	Spin-coating	Spin-coat a 7.5 µm thick SU-8 3005 layer on the wafer. On our device, we used 1500 rpm spinning speed for 30 sec.
4	Soft-bake	1 min at 65°C and subsequently 3 min at 95°C
5	UV-exposure	Exposure (i-line) with a light dose of 160 mJ mm^{-2} (measured intensity at 395 nm) through a foil mask.
6	Spin-coating	Spin-coat a 30 µm SU-8 3025 layer on the wafer. On our device, we used 3000 rpm spinning speed for 30 sec.
7	Soft-bake	2 min at 65°C and subsequently 10 min at 95°C
8	UV-exposure	Exposure (i-line) with a light dose of 160 mJ mm^{-2} (measured intensity at 395 nm) through a foil mask.
9	Post-exposure-bake	1 min at 65°C and subsequently 3 min at 95°C
10	Development	3-4 min in mr-Dev 600 under constant agitation, face-down
11	Hard bake	2 h at 160°C (ramp up in 40 min, ramp down slowly by switching the heater off)
12	HDMS coating	300 sec at 50 mbar
13	Spin-coating	Spin-coat a AZ 40XT layer (25 µm final height) on top of the existing structures (2300 rpm for 20 sec). Use excess resist to cover all structures before the spinning process is started. This prevents from inclusion of air and bubble formation.
14	Layer relaxation	To create smooth transitions between the AZ layer and the protruding SU-8 pillars (future magnetic traps), place the spin-coated wafer on an even surface for 10min.
15	Soft-bake	7 min at 85°C and subsequently 5 min at 120°C
16	UV-exposure	Exposure (i-line) with a light dose of 450 mJ mm^{-2} (measured intensity at 395 nm) through a foil mask.
17	Post-exposure bake	1 min at 85°C and subsequently 2 min at 105°C
18	Development	3 min in AZ 400K developer under constant agitation, face-down
19	Reflow	1 min at 115°C
20	Silanization	Place the final wafer for at least 24 h in a dessicator at 300 mbar together with 200 µL trichloro(1H,1H,2H,2H-perfluorooctyl)silane
21	Silanization	Place the wafer for another 2h in a desiccator at 300 mbar together with 200 µL chlorotrimethylsilane
22	PTFE-coating	Spin-coat the wafer at 500 rpm for 30 sec with a 0.1% Teflon AF solution in fluorinated FC-40 oil.
23	Oil evaporation	Evaporate residual oil by heating the wafer to 100°C for 5 min.

Table S4: Fabrication protocol for the double-layer PDMS chips. As a prerequisite, the two silicon master molds have to be fabricated beforehand (multilayer fluid master fabrication is described in Table S1, the control layer master consists of a single 20 µm SU-8 3025 structure).

Step	Name	Details
1	Mix PDMS	Mix 60 g PDMS monomer and curing agent at a ratio of 10:1.
2	Degas PDMS	To remove gas from the PDMS mixture, place the mixture in a desiccator for 15 min under vacuum.
3	Cast PDMS onto fluid master	Pour 40 g PDMS onto the fluid layer master that has been placed in a plastic petri dish to a final thickness of approximately 4 mm.
4	Store PDMS	To avoid PDMS hardening of the remaining PDMS, place it in the fridge at 4°C.
5	Bake fluid layer	Bake for 120 min at 80°C.

**6**	Cut fluid layer	Peel cured PDMS from the master and cut the chips to size using a razor blade.
**7**	Punch inlet and outlet ports	In- and outlet ports are punched with 1.5 mm biopsy punchers.
**8**	Spin-coat PDMS onto control layer	Use 5 g of the PDMS that was stored in the fridge and spin-coat the PDMS onto the control layer master for 60 sec at 2000 rpm to yield a homogeneous layer covering the control structures with a thin membrane.
**9**	Bake control layer	Place the control layer in the oven at 80°C for 60 min.
**10**	Prepare PDMS to PDMS bonding	Spin-coat 1 mL PDMS curing agent onto a blank silicon wafer at 6000 rpm for 40 sec.
**11**	Dip-coat curing agent	Dip the prepared PDMS slabs (fluid layer cut to size and with punched ports) onto the blank wafer that is covered with a thin curing agent layer.
**12**	Align PDMS parts	Detach PDMS slab from the blank wafer and align it to the control structures on the second wafer with the spin-coated PDMS layer. Redo steps 11 and 12 for all individual chips on the control layer. Finally pour remaining PDMS around the chips.
**13**	Diffusion	Let the wafer with the aligned chips sit on the bench for 30 min to allow diffusion of the curing agent.
**14**	Bake chips	Bake the PDMS composite for 60 min at 80°C.
**15**	Cut chips	Carefully peel the chips from the control master and punch control ports with a 1 mm biopsy puncher.
**16**	Clean glass slides	Clean glass cover slips with isopropanol, ethanol, water, and place them on a hotplate at 80°C for 10min.
**17**	Plasma bonding	Plasma activate the glass substrate as well as the chip in air plasma at 0.75 mbar for 40 sec and bond the chips to the glass.
**18**	Bake the composite chip	To increase the bonding strength, place the chip onto a hot plate at 80°C for 20 min.
**19**	Storage	Store the chips until use in the fridge at 4°C. This prevents from continuous hardening of the chips.

Table S5: Hardware of the three microscopes used for this project. The Nikon Ti and Ti2 microscopes were set up similarly in different biosafety levels to allow for similar conditions for tests over extended periods. The Olympus system is equipped with a high sensitivity EmCCD camera and was used to monitor the passage of missed cells and determine the overall capture efficiency.

**Microscope**	**Olympus X71**	**Nikon Ti**	**Nikon Ti2**
**Tests**	Capture efficiency	GCSF-calibration<br>Cell lines tests<br>Patient samples	Viability tests<br>Bead capture test
**Camera**	Andor iXon Ultra	Hamamatsu orca flash	Hamamatsu orca flash
**Objectives**	10×, NA=0.45	20×, NA=0.75	20×, NA=0.75
**Channels**	BF, GFP	BF, DAPI, GFP, Cy3, Cy4.9, Cy5.5, perCP	BF, DAPI, GFP, Cy3, Cy4.9, Cy5.5, perCP
**LED light source**	SpectraX	SpectraX	SpectraX
**Temperature control**	None	Live imaging solutions – the block	Live imaging solutions – the block
**Humidity control**	None	Live imaging solutions – the brick	Live imaging solutions – the brick

Table S6: Optical configurations.

Channel	Excitation filter	Emission filter	Targeted label
**Brightfield**	White light	White light	-
**DAPI**	390/18	460/50	NucBlue
**GFP**	475/28	535/50	HER2
**Cy3**	549/15	593/40	G-CSF
**Cy4.9**	390/18	670/30	CD45
**Cy4.9**	632/22	670/30	EpCAM/Barcode 1
**Cy5.5**	632/22	725/40	Barcode 2

Supplementary videos.

Supplementary Video SV1: Binding of target molecules in the microchamber over time. COMSOL simulation.

Supplementary Video SV2: Failed capture of a CTC cluster at a flow rate of 100 μL min^{-1}. Optimal cell capture efficiencies were seen at a maximum flow rate of 20 μL min^{-1}. Imaged with a 20× NA=0.75 objective and a Hamamatsu Orca Flash camera.

Supplementary Video SV3: Capture of a CTC on the chip. Imaged with a 20× NA=0.45 objective and an Andor iXON Ultra camera.

Supplementary file.

AutoCAD drawing of the microfluidic device.
